# The Predictive Role of Parathyroid Hormone for Nonalcoholic Fatty Liver Disease following Bariatric Surgery

**DOI:** 10.1155/2022/7319742

**Published:** 2022-02-28

**Authors:** Tannaz Jamialahmadi, Mohsen Nematy, Mohammed Abdalla, Ali Jangjoo, Ladan Goshayeshi, Matthew Kroh, Seyed Adel Moallem, Mitra Abbasifard, Thozhukat Sathyapalan, Amirhossein Sahebkar

**Affiliations:** ^1^Surgical Oncology Research Center, Faculty of Medicine, Mashhad University of Medical Sciences, Mashhad, Iran; ^2^Academic Diabetes, Endocrinology and Metabolism, Hull York Medical School, University of Hull, Hull, UK; ^3^Surgical Oncology Research Center, Imam Reza Hospital, Faculty of Medicine, Mashhad University of Medical Sciences, Mashhad, Iran; ^4^Department of Gastroenterology and Hepatology, Faculty of Medicine, Mashhad University of Medical Sciences, Mashhad, Iran; ^5^Gastroenterology and Hepatology Research Center, Mashhad University of Medical Sciences, Mashhad, Iran; ^6^Digestive Disease and Surgery Institute, Cleveland Clinic Lerner College of Medicine, Cleveland, OH, USA; ^7^Department of Pharmacology and Toxicology, College of Pharmacy, Al-Zahraa University for Women, Karbala, Iraq; ^8^Department of Pharmacodynamics and Toxicology, School of Pharmacy, Mashhad University of Medical Sciences, Mashhad, Iran; ^9^Immunology of Infectious Diseases Research Center, Research Institute of Basic Medical Sciences, Rafsanjan University of Medical Sciences, Rafsanjan 7718175911, Iran; ^10^Department of Internal Medicine, Ali-Ibn Abi-Talib Hospital, School of Medicine, Rafsanjan University of Medical Sciences, Rafsanjan 7718175911, Iran; ^11^Applied Biomedical Research Center, Mashhad University of Medical Sciences, Mashhad 9177948564, Iran; ^12^Biotechnology Research Center, Pharmaceutical Technology Institute, Mashhad University of Medical Sciences, Mashhad 91177948954, Iran; ^13^Department of Medical Biotechnology and Nanotechnology, Faculty of Medicine, Mashhad University of Medical Sciences, Mashhad 9177948954, Iran; ^14^Department of Biotechnology, School of Pharmacy, Mashhad University of Medical Sciences, Mashhad 9177948954, Iran

## Abstract

**Background:**

Morbid obesity is frequently complicated by chronic liver diseases, including nonalcoholic fatty liver disease (NAFLD), nonalcoholic steatohepatitis (NASH), and fibrosis. Parathyroid hormone (PTH) is found to be elevated in morbid obesity due to the defective hepatic metabolism of vitamin D. Bariatric surgery is performed to help patients with BMI>40 kg/m^2^ to effectively lose weight, particularly in patients with obesity who are afflicted with complications such as NAFLD/NASH.

**Objective:**

This study aimed to evaluate the PTH level as a predictor of hepatic function in individuals with morbid obesity who have undergone bariatric surgery.

**Methods:**

Ninety subjects with morbid obesity referred for Roux en-Y gastric bypass surgery were recruited. After IRB approval, demographic profiles, anthropometric factors, liver biopsy, and laboratory tests were obtained. The two-dimensional shear wave elastography (2D-SWE) technique was applied to assess hepatic stiffness.

**Results:**

A significant reduction occurred six months after bariatric surgery in the anthropometric indices (*p* < 0.001), hepatic elasticity (*p*=0.002), alanine aminotransferase (*p* < 0.001), serum alkaline phosphatase (*p* < 0.001), gamma-glutamyl transpeptidase (GGT) (*p* < 0.001), and nonalcoholic fatty liver disease fibrosis score (NFS) (*p* < 0.001). Serum PTH concentration was not predictive of postsurgical liver fibrosis and steatosis at six months but could predict weight loss success rate. No significant alteration in serum PTH levels was observed between presurgical vs. postsurgical time points.

**Conclusion:**

A significant reduction was observed in the anthropometric parameters, liver enzymes, and hepatic elasticity after bariatric surgery. No significant effect was found on PTH levels.

## 1. Introduction

Worldwide, along with a rapid rise in the number of patients with obesity, nonalcoholic fatty liver disease (NAFLD) prevalence is also increasing, affecting 20–30% of the population across the world [1]. Considering the potential of obesity as the most common risk factor for developing NAFLD, approximately 90% of adults with morbid obesity have NAFLD. This prevalence is higher with other weight-related comorbidities such as type 2 diabetes mellitus (T2DM) [2]. Another contributor to the formation of NAFLD is assumed to be vitamin D deficiency, which is also related to the development of fibrogenesis and insulin resistance (IR) [3, 4]. However, the downstream effects of vitamin D on NAFLD are not yet clarified. It is uncertain whether calcium (Ca), activated 1*α*, 25-dihydroxy vitamin D3 (1*α*, 25 (OH) 2D3), 25-hydroxy vitamin D3 (25 (OH) D3), or parathyroid hormone (PTH) can also predict disease progression. Additionally, liver disease progression can impair vitamin D metabolism [5, 6]. Furthermore, patients with morbid obesity and vitamin D deficiency (VDD) may be at a higher mortality risk due to hepatic dysfunction [7, 8].

Bariatric and metabolic surgeries are procedures that effectively facilitate and maintain weight loss. Even 10% of total body weight loss following surgery is shown to improve liver steatosis, nonalcoholic steatohepatitis (NASH), and reverse hepatic fibrosis [9]. A significant drawback of bariatric surgery can be the malabsorption of calcium and vitamin D, leading to low circulating calcium levels and a subsequent rise in the parathyroid hormone (PTH) [10]. In order to avoid calcium and vitamin D deficiencies, the intake of supplements containing those nutrients is routinely recommended, especially after malabsorptive procedures such as Roux en-Y gastric bypass [11].

One of the more common adverse effects of bariatric surgery is malnutrition, which can negatively affect the mineralization of bones and set the stage for fracture formation. A low level of vitamin D can cause long-term secondary hyperparathyroidism (SHPT) [6, 12]. There is scarce evidence showing that the parathyroid hormone level among individuals with morbid obesity remains elevated before and after surgery, particularly in patients who underwent one-anastomosis gastric bypass (OAGB) and Roux en-Y gastric bypass [13–15]. This study focused on the plausible role of PTH in the prediction of NAFLD in patients with obesity following bariatric surgery.

## 2. Materials and Methods

### 2.1. Participants

Based on our previous study, patients who had BMI more than 40 kg/m^2^ or BMI between 35 and 40 kg/m^2^ with two or more comorbidities undergoing Roux en-Y gastric bypass were recruited. This study was carried out in Mashhad, Iran, from Dec 2016 to Sep 2017 on 90 consecutive individuals with morbid obesity (age range: 18–67) referred to the outpatient clinic of Imam Reza Teaching Hospital for gastric bypass surgery. All participants completed informed consent. Exclusion criteria were both alcohol use exceeding 30 g/day in males and 20 g/day in females, chronic liver disease caused by medication, hepatitis B (HBs) Ag, and hepatitis C virus (HCV) positive antibodies. All of the procedures had been approved according to the ethical standards of the institutional and national research committee and with the 1964 Helsinki Declaration and its later amendments or comparable ethical standards. The classification of metabolic syndrome is according to the National Cholesterol Education Program expert panel on detection, evaluation, and treatment of high blood cholesterol in adults (NCEP ATP III).

### 2.2. Laboratory Tests

Fasting blood samples were drawn from all participants to assess laboratory parameters, including a lipid profile, liver enzymes including aspartate aminotransferase (AST, U/L), gamma-glutamyl transferase (GGT, U/L), alkaline phosphatase (ALP, U/L), and alanine aminotransferase (ALT, U/L), glycemic status (fasting blood sugar (mg/dL), fasting insulin (*μ*IU/L), HOMA-IR index (homeostasis model assessment insulin resistance), and (serum level of PTH).

### 2.3. Anthropometric Indices

The standard protocol is used to evaluate anthropometric indicators, including height, waist circumference, weight, and BMI. Body analysis (body fat mass, body fat percentage, and fat-free mass) was done by a bioelectrical impedance analyzer, Tanita BC-418 (Tanita Corp., Tokyo, Japan).

### 2.4. Two-Dimensional Shear Wave Elastography

Liver stiffness measurement was done in left lateral decubitus, while the right arm was abducted maximally in 6 h fasted patients by 2D-SWE. The measurement was carried out by a curved broadband probe (SC6-1, 1–6 MHz) using the Aixplorer ultrasound system (Supersonic Imagine, France). Ideal liver stiffness result (LSM) was according to the mean image acquisitions of everyone. A blinded operator reported the mean (*M*) of ten valid LSM (liver stiffness measurement) in kilopascals (kPa).

### 2.5. Gastric Bypass Surgery

Roux en-Y gastric bypass performed laparoscopically antecolic and antegastric resulted in a biliopancreatic limb (approximately 70 cm) and a Roux limb (100–150 cm) to create a vertically oriented gastric pouch (30–50 cc). A three-row stapler was used to create end-to-side gastrojejunostomy and side-to-side jejunojejunostomy. Directly visualized liver biopsies of the left hepatic lobe were extracted during surgery by a 16-gauge Tru-Cut needle.

### 2.6. Postsurgery Follow-Up

All the participants were reevaluated six months postoperation regarding anthropometric indices (body fat percentage, waist circumference, body mass index (BMI), and fat-free mass) and metabolic, liver, and inflammation panel (liver function tests (LFT), lipid, and glycemic profile), and 2D-SWE.

### 2.7. Success Rate

Excess weight loss (EWL) of more than 50% six months after bariatric surgery was considered successful weight loss. The formula used to calculate % EWL was (weight loss/baseline excess weight) × 100, where weight loss = preoperative weight − postoperative weight; baseline excess weight = initial weight minus the ideal weight [16]. The percentage of total weight loss (%TWL) was calculated by division of preoperative weight − follow-up weight by the preoperative weight and multiplying by 100. ((Preoperative BMI−current BMI)/(preoperative BMI−25)) ×10 was used to estimate the percentage of excess BMI loss (%EBMIL) (37).

### 2.8. FIB−4

FIB−4 = age (years)×AST (U/L)/platelet count (109/L) × √ALT(U/L).

### 2.9. NFS

NFS = −1.675 + 0.037 × age (years) + 0.094 × body mass index (kg/m^2^) + 1.13 × (impaired fasting glycemia or diabetes (yes = 1, no = 0)) + 0.99 × (AST/ALT ratio) − 0.013 × platelets (×109/L) − 0.66 × albumin (g/dL).

### 2.10. Statistical Analysis

The description of parametric and nonparametric values was as mean ± standard deviation (SD) and median (interquartile range (IQR)), respectively. Spearman's coefficient was applied to represent the relationship between the data. SPSS (version 25) was used for statistical analysis. A regression model was utilized to predict the relationship between PTH as a predictor and postoperation fatty liver disease and success rate. If appropriate, the *p* value for all statistical tests was considered significant at the threshold of 5%.

## 3. Results

### 3.1. Demographic Details

The mean age of the 90 patients involved in the study was 38.5 ± 11.1 years, with 72 (80%) females. Also, the mean BMI was 45.46 ± 6.26 kg/m^2^, and the mean body weight was 121.34 ± 20.32 kg. Metabolic syndrome affected more than half (51.9%) of the participants (considered as the existence of at least three of five to be followed criteria: waist circumference more than 102 cm (male) and 88 cm (female), high blood pressure (>130/85 mmHg), fasting high-density lipoprotein (HDL) cholesterol lower than 40 mg/dL (male) or 50 mg/dL (female), fasting triglyceride (TG) levels more than 150 mg/dL, and fasting blood glucose higher than 110 mg/dL) by the criteria of the National Cholesterol Education Program expert panel on detection, evaluation, and treatment of high blood cholesterol in adults (NCEP ATP III) [17]. As given in Table 1, the mean liver elasticity evaluated through 2D-SWE preoperatively was 6.1 ± 1.25 kPa.

### 3.2. Anthropometric Status Pre and Postsurgery

The average weight loss was 33.93 ± 11.79 kg ranging from 25.31 ± 9.40 kg to 11.88 ± 12.86 kg. Also, the anthropometric profile was significantly reduced after surgery (*p* < 0.001) (Table 2). Six months postoperatively, the mean % total weight loss (%TWL) was calculated as 27.96 ± 6.43% in our population, ranging from 11.47% to 47.75%. In general, postsurgery excess body mass index loss (EBMIL%) and excess weight loss (EWL%) were 63.70 ± 15.27% and 63.92 ± 14.66%, respectively.

### 3.3. Liver Status and PTH Level Pre and Postsurgery

On average, there was a significant reduction in liver elasticity from 6.1 ± 1.25 to 5.42 ± 1.52 kPa (*p*=0.002). Also, there was a reduction in liver aminotransferases; for alanine aminotransferase, this reached a significant level (*p* value < 0.001). Furthermore, serum levels of alkaline phosphatase significantly increased (196.25 ± 53.79–222.50 ± 65.61 (IU/L)) (*p* value < 0.001). The level of GGT had a significant reduction from 31.62 ± 20.21 to 20.63 ± 24.79 (IU/L) (*p* value < 0.001). FIB-4 index reduction was not significant. Also, the nonalcohol fatty liver disease score (NFS) was reduced significantly from −1.42 ± 1.21 to −2.26 ± 1.25 (*p* < 0.001). Steatosis grade was also changed significantly. Platelet count was decreased by 22.96 ± 60.45 (n/*μ*L) (*p* value < 0.001). Moreover, circulating PTH levels reduced nonsignificance from 47.88 ± 24.22 to 45.44 ± 25.94 (Table 3).

### 3.4. Changes in PTH and Liver Status Postbariatric Surgery and Their Relationship

The relationship between the circulating PTH level and liver fibrosis (elastography), steatosis (ultrasonography), success rate, FIB-4, and NFS is given in Table 4. Positive correlations of PTH with steatosis (evaluated via ultrasonography), liver fibrosis (measured by elastography), FIB-4, and NFS were not significant. Also, a negative nonsignificant correlation was observed between PTH and success rate.

### 3.5. Regression Analysis between PTH before Surgery and Postoperation Liver Parameters

Binary logistic regression analysis was studied for PTH with postoperation success rate and fibrosis (elastography) after adjusting by gender, baseline WC, AST, GGT, ALT, age, ALP, and HOMA-IR. Based on regression analysis, the circulating PTH level was a significant predictive factor for success rate in the adjusted model. Ordinal regression analysis for PTH with postoperation steatosis (ultrasonography) was studied after adjusting by baseline WC, age, ALP, gender, AST, GGT, ALT, and HOMA-IR on subject groups. Linear regression analysis for PTH with postoperation FIB-4 was studied after adjusting by gender, baseline ALP, WC, GGT, and HOMA-IR on subject groups. Linear regression analysis for PTH with postoperation NFS was studied after adjusting by gender, baseline GGT, and ALP on subject groups (Table 5).

According to the regression analysis, the level of circulating PTH was not a predictive factor for liver status in both adjusted and unadjusted models.

## 4. Discussion

In this study, there was a significant reduction in markers of adiposity after bariatric surgery. Furthermore, a significant reduction of alanine aminotransferase also occurred. Although the elevation of serum alkaline phosphatase was significant, PTH levels remained unchanged. Also, we found that the serum PTH level was a significant predictive factor for weight loss success rate after bariatric surgery.

Obesity can be associated with high PTH levels resulting from vitamin D deficiency because vitamin D intake by adipose tissue is increased, sunlight exposure is limited due to inactivity, and vitamin D production in the liver is diminished due to a functionally compromised liver affected by steatosis. Bariatric surgery has been shown to reduce adipose tissue mass while also improving obesity-associated comorbidities [13]. Although bariatric surgery is a successful treatment for morbid obesity, it decreases calcium absorption, resulting in a substantial decrease in bone density and, as a result, an increase in PTH levels [13]. The elevated levels of PTH before bariatric surgery may be attributed to a vitamin D deficiency caused by NAFLD, which may be exacerbated by postbariatric surgery calcium absorption impairment [18].

A previous study showed that an increased level of PTH is a predictive factor for nonalcoholic steatohepatitis (NASH), especially in patients with morbid obesity who were candidates for bariatric surgery [19]. Moreover, PTH levels were significantly higher in patients with pathological and imaging evidence of steatosis, NASH, and hepatic fibrosis [20].

Although there was an increase in ALK-P after surgery as a sign of bone resorption, perioperative PTH levels remained unchanged. On the other hand, it should be noted that a six-month follow-up is relatively short to detect changes in bone turnover markers and hepatic function. Consequently, the PTH level was not able to predict hepatic function postoperatively.

This current study shows that the preoperative PTH level has an excellent predictive value for success in weight loss after surgery. It is noteworthy that pre and post-dysregulation of the PTH axis may alter and even limit total weight loss (TWS) after surgery. In a study by Alexandro et al., patients with a higher TWS were shown to be less likely to have PTH dysregulation, which is consistent with our findings [21]. Interestingly, considering the impact of PTH on the bone and kidney, it is suggested that hyperparathyroidism promotes weight gain by increasing intraadipocyte free calcium, thus blunting the lipolytic reaction to catecholamines [22]. Furthermore, given that weight alteration is not only because of fat mass but also lean mass and muscle mass may be affected, it is necessary to consider extensive evaluation of comorbidities of metabolic surgeries rather than focus just on weight.

In a previous study published by our group, we demonstrated that high levels of PTH were significantly associated with histological indices of hepatic fibrosis, steatosis, and NASH in patients with morbid obesity before surgery. This may appear to be in contradiction to the current findings. Previous study, however, was a cross-sectional study design which does not establish causality [15]. In this current longitudinal study, we evaluated the chronological framework between the status of liver fibrosis and elevated levels of PTH. In one sense, PTH levels would be expected to decrease because weight loss causes PTH levels to reduce in morbidly obese people [23]. On the other hand, postoperative secondary hyperparathyroidism (SHP) can occur as a result of vitamin D malabsorption following surgery [12]. Another significant challenge encountered during the study was the unknown sun exposure of individuals following surgery [12]. As a result, serum PTH levels are affected by a variety of factors, which should be interpreted in light of preexisting abnormalities in obese patients.

Due to this finding, we suggest identifying these risk factors before surgery and discussing the potential applications for this procedure. Further investigation and potential intervention are recommended in high-risk patients.

## 5. Conclusion

In this study, prebariatric surgery PTH levels did not correlate with improved liver disease status following surgery. Therefore, PTH and NAFLD association might be independent of weight. These findings may be due to a short follow-up period in our study, which necessitates more long-term evaluations in future.

## Figures and Tables

**Table 1:** Patient characteristics.

Variable	Total
Male, *N* (%)	18 (20)
Age (year)	38.5 ± 11.1
BMI (kg/m^2^)	45.46 ± 6.26
Weight (kg)	121.34 ± 20.32
Waist circumference (cm)	133.04 ± 13.6
Height (m)	1.62 ± 8.87
Diabetes type 2	25 (27.8)
Hypertension	23 (25.6)
Metabolic syndrome	46 (51.1)

Data mentioned in *N* (%), mean ± SD. BMI, body mass index.

**Table 2:** Anthropometric indices in clinically severe obese patients before and after bariatric surgery.

Variable	Before	After	Changes	*P* value
BMI (kg/m^2^)	45.46 ± 6.26	32.41 ± 4.47	−13.05 ± 3.85	<0.001
Weight (kg)	121.34 ± 20.32	87.41 ± 14.01	−33.93 ± 11.79	<0.001
Waist circumference (cm)	133.04 ± 13.6	107.61 ± 1.52	−25.43 ± 12.13	<0.001
Body fat percentage (%)	25 (27.8)	35.83 ± 7.63	−10.88 ± 4.77	<0.001
Fat-free mass (kg)	23 (25.6)	52.62 ± 15.16	−11.88 ± 12.86	<0.001

Data mentioned in *N* (%), mean ± SD; BMI, body mass index.

**Table 3:** Liver status and PTH level in clinically severe obese patients before and after bariatric surgery.

Variable	Before	After	*P* value
Liver stiffness measurement (kPa)	6.1 ± 1.25	5.42 ± 1.52	0.002
AST (IU/L)	23.60 ± 10.48	22.26 ± 12.93	0.382
ALT (IU/L)	26.69 ± 19.30	19.92 ± 11.79	<0.001
GGT (IU/L)	31.62 ± 20.21	20.63 ± 24.79	<0.001
ALP (IU/L)	196.25 ± 53.79	222.50 ± 65.61	<0.001
FIB-4	0.59 ± 0.26	0.57 ± 0.40	0.123
NFS	−1.42 ± 1.21	−2.26 ± 1.25	<0.001
Steatosis (ultrasonography)			<0.001
Grade 0	5 (5.5)	16 (18)	
Grade 1	19 (21.1)	47 (52.8)	
Grade 2	53 (58.8)	24 (27)	
Grade 3	13 (14.4)	2 (2.2)	
PTH (pg/mL)	47.88 ± 24.22	45.44 ± 25.94	0.464

Data mentioned in *N* (%), mean ± SD; AST, aspartate aminotransferase; ALT, alanine aminotransferase; ALP, alkaline transferase; GGT, gamma-glutamyl transferase; FIB-4, fibrosis 4; NFS, NAFLD fibrosis score; PTH, parathyroid hormone.

**Table 4:** Relationship between baseline PTH and liver status after bariatric surgery.

	PTH	
CC	*r*	*P* value
FIB-4	0.111	0.333
NFS	0.163	0.186
Success rate	−0.173	0.125
Fibrosis (elastography)	0.116	0.304
Steatosis (ultrasonography)	0.081	0.466

PTH, parathyroid hormone; CC, correlation coefficient; FIB-4, fibrosis 4; NFS, NAFLD fibrosis score.

**Table 5:** The logistic regression analysis between PTH and study parameters.

Parameters		*P*	OR	95% CI for OR	
				Lower	Upper
Crude model	FIB-4	0.914	−0.001	−0.013	0.012
	NFS	0.967	0.001	−0.041	0.043
	Success rate	0.127	0.987	0.971	1.004
	Fibrosis (elastography)	0.946	−0.002	−0.049	0.045
	Steatosis (ultrasonography)	0.541	1.005	0.990	1.020
Adjusted model	FIB-4^*∗*^	0.857	0.000	−0.004	0.005
	NFS^*∗∗*^	0.118	0.012	−0.003	0.027
	Success rate	0.025	0.971	0.946	0.996
	Fibrosis (elastography)	0.353	0.008	−0.009	0.026
	Steatosis (ultrasonography)	0.795	1.00	0.98	1.02

FIB-4, fibrosis 4; NFS, NAFLD fibrosis score. ^*∗*^Adjusted for sex: WC, HOMA-IR, GGT, Alp. ^*∗∗*^Adjusted for sex: GGT, Alp.

## Data Availability

The data used to support the findings of this study are available from the corresponding author upon request.
